# Evaluation of colon mucosa using screening colonoscopy and flexible spectral imaging color enhancement in patients with long lasting ulcerative colitis

**DOI:** 10.3325/cmj.2021.62.435

**Published:** 2021-10

**Authors:** Seda Akkaya Özdi̇nç, Hale Akpinar, Göksel Bengi, Sülen Sarioğlu, Özgül Sağol, Gozde Dervis Hakim, Mesut Akarsu, Müjde Soytürk, Omer Selahattin Topalak

**Affiliations:** 1 Department of Gastroenterology, Dokuz Eylul University Faculty of Medicine, İzmir, Turkey; 2 Department of Pathology, Dokuz Eylul University Faculty of Medicine, İzmir, Turkey; 3 Department of Gastroenterology, Tepecik Education and Research Hospital, İzmir, Turkey

## Abstract

**Aim:**

To determine which flexible spectral imaging color enhancement (FICE) channel best visualizes colon mucosa in ulcerative colitis (UC) and to compare FICE imaging with standard imaging.

**Methods:**

The study enrolled patients with ulcerative colitis in remission who had inflammatory bowel disease for at least 8 years. All patients underwent screening colonoscopy. The entire colon, especially the suspicious areas in terms of dysplasia, were imaged with standard endoscopy and FICE. Random and target biopsies were obtained. Histopathological diagnosis was made and image patterns were evaluated. Seven endoscopists evaluated normal, colitis, and polyp images obtained with FICE.

**Results:**

One hundred and twenty-three colon segments were evaluated and 1831 images were obtained from 18 patients. A total of 1652 images were FICE and 179 were standard images. Separate FICE images were obtained for normal colon mucosa, polypoid lesions, and colitis areas. Normal colon mucosa was best visualized using the second, sixth, and ninth FICE channel; polyps using the third, seventh, and ninth channel; and colitis using the second, third, and ninth channel. When all images were analyzed, the second and ninth channel were significantly better than the other channels. A total of 584 biopsies were obtained, including 492 (84.2%) random biopsies and 92 (15.7%) target biopsies. Random biopsies detected no dysplasia, but target biopsies detected low-grade dysplasia in three diminutive polyps.

**Conclusion:**

FICE was not significantly better at dysplasia screening than the standard procedure, but it effectively detected diminutive polyps and evaluated surface patterns without using magnification. FICE might contribute to the assessment of inflammation severity in patients with UC in clinical remission. However, more extensive studies are necessary to confirm these findings.

In patients with ulcerative colitis (UC), intraepithelial neoplastic changes occurring as a result of prolonged chronic inflammation may lead to the development of colorectal cancer (CRC) (1). Patients with inflammatory bowel disease have a six times higher risk of CRC than the general population (2). A meta-analysis by Eaden et al showed that in patients who had UC for 20 years the risk of CRC increased by 8.5% and in those who had UC for 30 years the risk increased by 17.8% (3). The frequency of multiple synchronized malignancies was higher when CRC developed due to UC compared with sporadic CRC. Furthermore, CRC developing as a result of UC is often characterized by flat lesions.

To reduce the risk of CRC development in UC patients, current guidelines recommend the initiation of colonoscopy screening 8-10 years after the onset of symptoms. The risk is higher in patients with a family history of CRC, pancolitis, accompanying primary sclerosing cholangitis, early-age diagnosis, backwash ileitis, or severe inflammation; all of these patients are recommended to undergo screening. Early screening colonoscopy in UC has decreased the mortality from UC-associated cancers (4-6). The aim of screening colonoscopy is to search for suspicious mucosal lesions in terms of dysplasia. If no lesion is detected, biopsies from all four colon quadrants are obtained at 10-cm intervals (3). However, this method is of limited success since less than 0.05% of the colon is sampled. Moreover, flat lesions can cause sampling errors (7). Obtaining an increased number of samples from the colon has not increased the early-detection rate (8), although it prolongs the procedure time. Dysplastic and early neoplastic lesions are usually small, flat, and depressed, and therefore more likely to be overlooked with standard endoscopy. Instead of screening colonoscopy, new endoscopic imaging techniques are considered for target biopsies, especially for lesion detection. A new endoscopic imaging technique, chromoendoscopy (CE), identifies intraepithelial neoplasms 3 to 4.5 times better than the standard methods (9). On the other hand, it is difficult to stain the entire colon, and the method has reduced efficacy since inadequate colon cleansing can lead to false positive findings of dysplasia in the regions with severe inflammation (10). Better CE techniques are now developed, such as narrow-band imaging (NBI), flexible spectral imaging color enhancement (FICE), and I-scan. FICE allows a detailed examination of the mucosal structure by using selective wavelengths to transform the mucosa surface structure into newly arranged image structures through a digital image processing system (11).

FICE has not been used so far to evaluate dysplastic changes, especially in colon polyps and colorectal lesions (12). FICE with high-resolution magnifying endoscopy (HRME) showed high sensitivity and specificity in neoplastic differentiation of colon polyps according to surface patterns (13). However, to our knowledge, no studies have assessed the use of FICE in dysplasia screening in patients with UC. The aim of this study is to determine which FICE channel best visualizes colon mucosa in UC and to compare FICE imaging with standard imaging

## PATIENTS AND METHODS

### Patients

**Figure 1:**
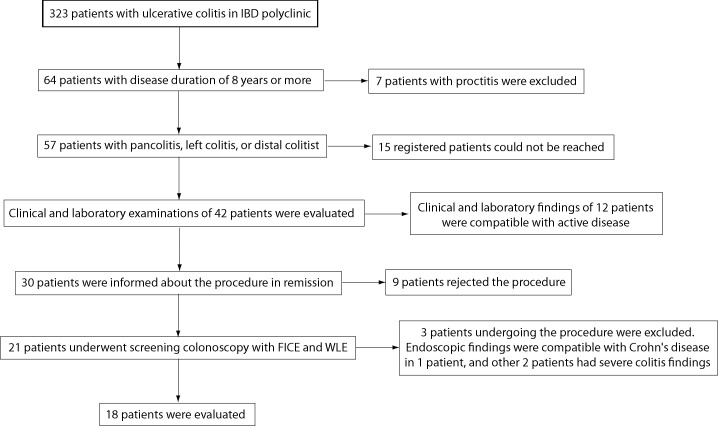
Patient group formation. IBD – ınflammatory bowel disease; FICE – flexible spectral imaging color enhancement; WLE – white light endoscopy.

The study enrolled 18 patients in UC remission who presented to Inflammatory Bowel Disease Polyclinic outpatient clinic with at least an eight-year follow-up after the onset of UC symptoms (Figure 1). The remission was clinically defined as stool count ≤3, no blood in stool, and C-reactive protein within the reference range. Patients younger than 18 years or older than 80 years, and those with active UC, severe comorbidity, or other conditions such as cardiovascular disease, chronic renal failure, and severe pulmonary disease were excluded. This study was approved by the Dokuz Eylul Medical Faculty Clinical and Laboratory Research Ethics Committee (2013/20-08).

### Colonoscopy

Colonoscopy was performed with a Fujinon EC-490ZW5 endoscope (Fuji Photo Optical Co. Ltd, Saitama, Japan). Before colonoscopy, standard bowel cleansing procedure was performed. All procedures were carried out under sedation. Standard images, FICE images taken with 10 different channels, and a “random” biopsy from each four-quadrant colon mucosa at every 10 cm going backward from the cecum were obtained. When a lesion was observed, standard imaging and 10-channels FICE imaging were repeated, and “target” biopsies for the lesion were obtained. The lesion shape and size were assessed according to the Paris classification, and the mucosal structure was evaluated with the Kudo pit pattern classification (14).

In total, during 18 colonoscopy procedures 179 standard images were taken. One hundred fifty FICE images were taken, ie, 50 images (5 images in 10 FICE channels) of each: normal colon mucosa, polyps, and inflamed colon mucosa. The images were scored by seven endoscopists during colonoscopy from 1 to 3, with 3 indicating the best image quality. The points of 10 FICE channels were summed up separately. Thus, a total score was obtained for each FICE channel. The images from two FICE channels with the highest score were compared with the standard image by seven endoscopists. The scores ranged between -1 and +3. The -1 score indicated that the FICE channel image was worse than the standard image, zero indicated no difference, and +3 meant that FICE was better than the standard image.

### Pathological evaluation

The biopsy samples were stained with hemotoxylin-eosin and evaluated by the same pathologist (SS) using light microscopy. The Vienna classification was used in dysplasia assessment (negative dysplasia, indeterminate dysplasia, and positive dysplasia) (15). In the presence of dysplasia, a second opinion was required.

### Statistical analysis

FICE channel total scores were calculated for normal, polyp, and inflamed colon mucosa, and analyzed as a sum. The scores are presented as a minimum, maximum, mean, standard deviation, median, and interquartile range (IQR). Non-parametric Friedman test was used for comparison of all channels. The Wilcoxon signed-rank test was used for paired comparisons. *P* < 0.05 was considered statistically significant. The data were analyzed with SPSS, version 16.0 (SPSS, Chicago, IL, USA).

## Results

After evaluating 323 patients with ulcerative colitis followed in the inflammatory bowel outpatient clinic, 18 patients who met the inclusion criteria were included in the study (Figure 1). Patients’ demographic data and medications are shown in Table 1.

**Table 1:** Demographic data and medications of ulcerative colitis patients

Parameter	
**Mean age ± standard deviation (min-max)**	**54.8 ± 14.2 (25-80)**
**Sex, n (%)**	
female	8 (44.4)
male	10 (55.5)
Mean disease duration ± standard deviation, years*	12.9 ± 3.6
**Disease involvement, n (%)**	
pancolitis	6 (33.3)
left colitis	6 (33.3)
distal colitis	6 (33.3)
**Medications, n (%)**	
mesalamine	11 (61.1)
mesalamine + azathioprine	4 (22.2)
azathioprine + infliximab	1 (5.5)
sulfasalazine	2 (11.1)
steroids at least once during the disease	10 (55.5)
**Extraintestinal findings, n (%)**	
arthritis	2 (11.1)

*The youngest age of onset: 13 years.

A total of 1831 images (an average of 101 ± 11 per patient) were taken for the evaluation of 123 colon segments in 18 patients. Overall, 1652 (90.2%) FICE images and 179 (9.7%) standard images were obtained. Images of normal colon mucosa, polypoid lesions, and endoscopically mild to moderate colitis areas were taken with FICE (Table 2). The biopsies results and duration of procedures are shown in Table 2.

**Table 2:** Characteristics of biopsies and images of ulcerative colitis patients

Parameter	
**Total number (%) of images;** mean ±SD per patient	
FICE	1652 (90.2); 91.7 ± 10
standard images	179 (9.8)
**Total number (%) of biopsies**	
random	492 (84.2)
targeted	92 (15.7)
**The mean duration of procedure in minutes (min-max)**	49.5 ± 8.5 minutes (35-66)

### FICE images of normal colon mucosa

**Figure 2:**
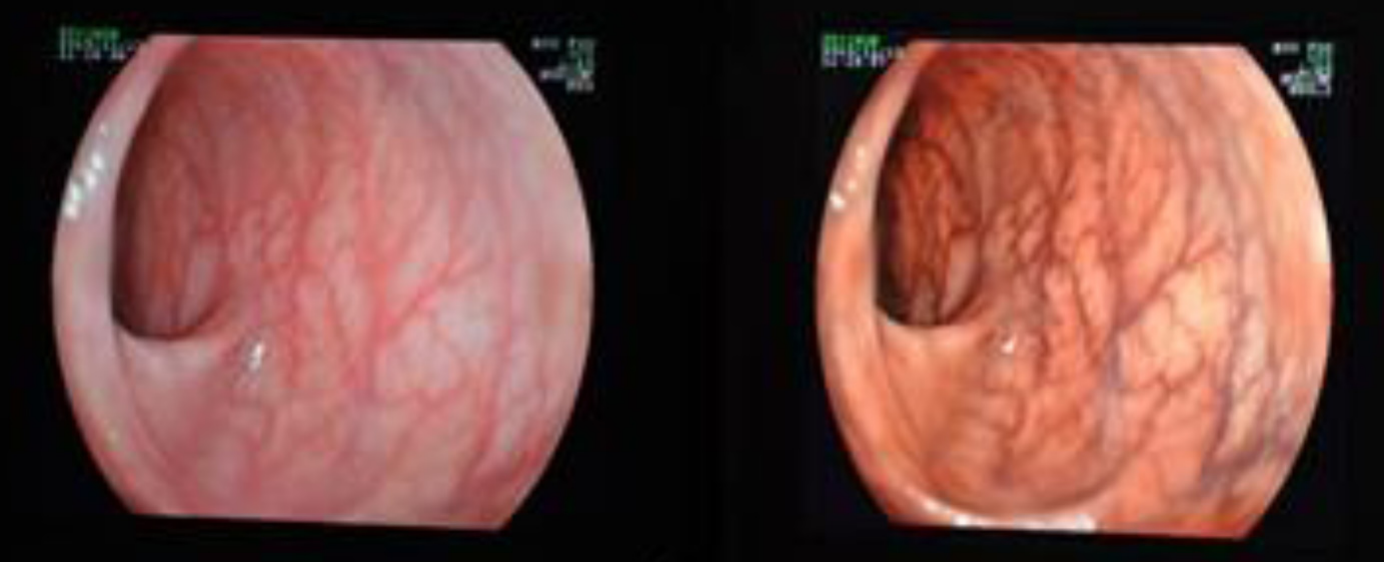
Normal colon mucosa (left) and the capillary network (right) (the second flexible spectral imaging color enhancement channel).

In FICE images of normal colon mucosa, capillary network was visible as light brown, while submucosal vessels were visible as dark brown. The surface structure and the capillary network were clearly distinguished (Figure 2).

The scores of 50 images of five patients with normal colon mucosa significantly differed across 10 FICE channels (*P* = 0.001) (Table 3). The channels that best demonstrated the mucosal vascular structure were the second, sixth, and ninth channel (*P* < 0.05). The highest median (IQR) values were observed in the second channel (14, IQR 4), followed by the sixth channel (11, IQR 3.5), and the ninth channel (11 IQR 4.5), without significant difference among the channels.

**Table 3:** Rating scores awarded to images obtained with different flexible spectral imaging color enhancement (FICE) channels for normal colon

Channels	Minimum	Maximum	Mean	Standard deviation	Median	Interquartile range	*P*
0	0	3	1	1.414	0	2.5	<0.001
1	0	5	2.6	1.817	3	3	
2	10	16	13.2	2.28	14	4	
3	0	4	1.4	1.517	1	2	
4	0	0	0	0	0	0	
5	0	2	0.6	0.894	0	1.5	
6	9	14	11.2	1.924	11	3.5	
**7**	0	2	0.8	0.837	1	1.5	
**8**	0	0	0	0	0	0	
**9**	**8**	**15**	**11.2**	**2.588**	**11**	**4.5**	

### FICE images of raised or polypoid lesions in the colon mucosa

**Figure 3:**
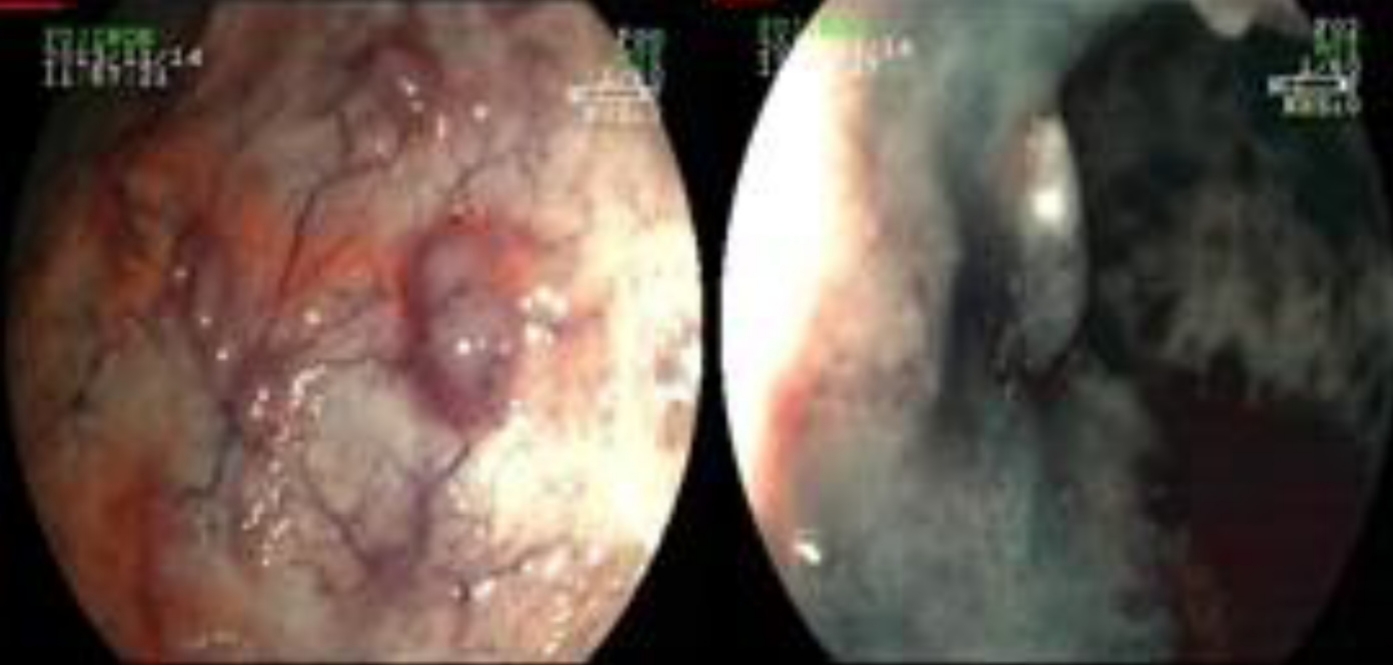
A tubular polyp with an oval pit pattern as depicted with the second flexible spectral imaging color enhancement (FICE) channel (left) and the fourth FICE channel (right).

A total of 16 polyps were detected (Table 4). One patient had two polyps smaller than 5 mm (Paris classification – Is): one in the ascending colon and one in the transverse colon. Tubular and oval pits presenting on the polyp surfaces were evaluated as neoplastic pattern (Figure 3). Histopathological examination of the biopsies revealed an adenomatous polyp and low-grade dysplasia. The surface of another polyp of 2 mm (Is) in the descending colon could not be assessed clearly, but the histopathological finding was similar.

**Table 4:** Characteristics of polyps detected in patients with ulcerative colitis

Parameter	N
**Size of polyps**	
<5 mm	4
5-10 mm	10
>10 mm	2
**Site**	
cecum	2
ascending colon	2
transverse colon	5
descending colon	2
sigmoid colon	2
rectum	3
**Structural features** **(Paris classification)**	
Is	13
Ip	2
IIa	1
**Pit pattern** **(Kudo pit pattern)**	N
non-neoplastic pattern (type i)	13
neoplastic pattern (type iii)	2
could not be assessed	1
**Pathological diagnoses**	
inflammatory polyp	11
hyperplastic polyp	2
low grade dysplasia in adenomatous polyp	3

**Figure 4:**
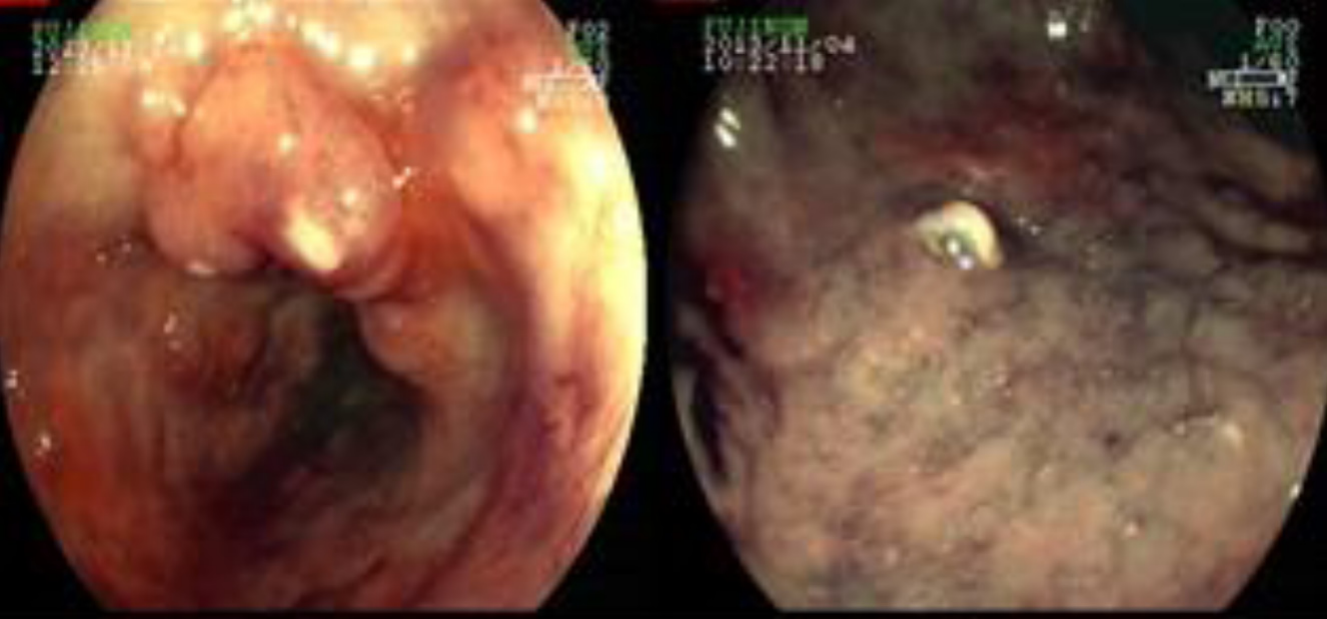
A round pit pattern polyp (post-inflammatory polyp) as depicted with the third flexible spectral imaging color enhancement (FICE) channel (left) and the zeroth FICE channel (right).

**Figure 5:**
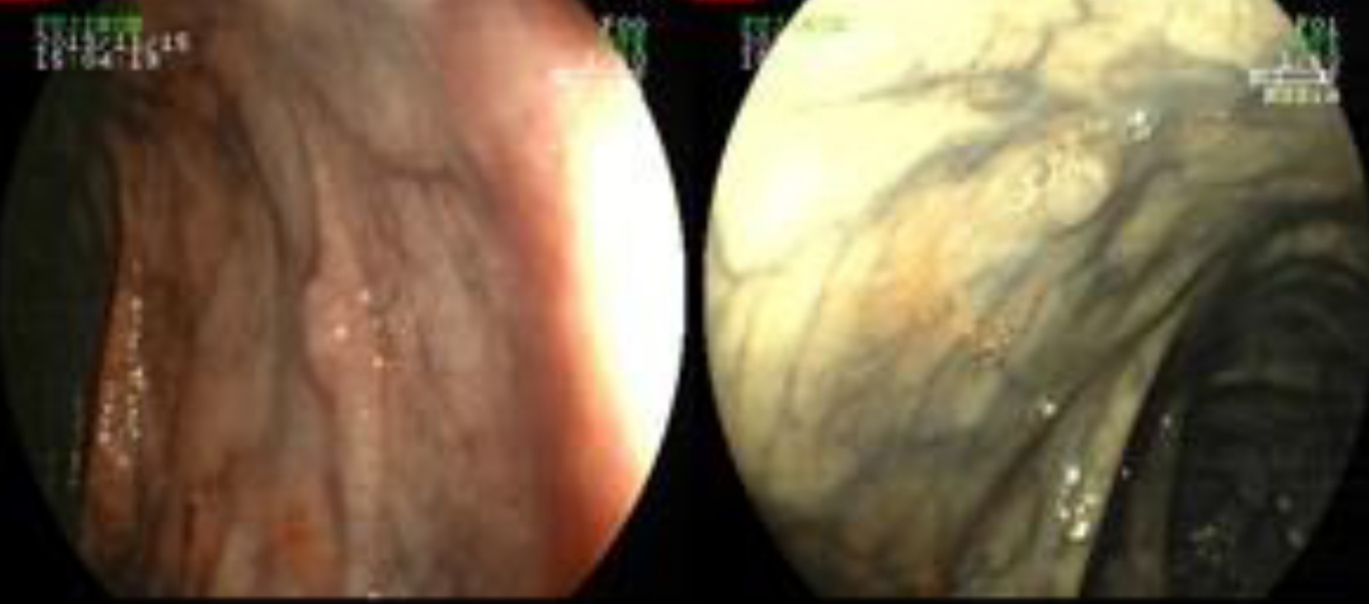
A round pit pattern polyp (post-inflammatory polyp) as depicted with the ninth flexible spectral imaging color enhancement (FICE) channel (left) and a diminutive polyp with a round pit pattern (post-inflammatory polyp) as depicted with the seventh FICE channel (right).

Of the ten polyps with round pit pattern, eight were histopathologically determined to be post-inflammatory polyps (Figure 4 and Figure 5), whereas two were hyperplastic polyps.

The scores of 50 images of five polyps significantly differed across 10 channels (*P* = 0.01). The median values in the third, seventh, and ninth channel were 5 (IQR, 3.5), 7 (IQR, 3), and 10 (IQR, 6.5), respectively. The median score of the ninth channel was significantly higher than that of the third channel (*P* = 0.42). There was no difference between the ninth and seventh channel or between the seventh and third channel (Table 5).

**Table 5:** Rating scores awarded to images obtained with different flexible spectral imaging color enhancement (FICE) channels for polyps

Channels	Minimum	Maximum	Mean	Standard deviation	Median	Interquartile range	*P*
0	0	9	4.6	4.159	3	8	0.010
1	3	11	5.4	3.578	3	6	
2	0	8	3.6	3.05	3	5.5	
3	1	6	4.4	2.074	5	3.5	
4	0	5	2.2	1.924	2	3.5	
5	0	2	0.8	1.095	0	2	
6	1	7	3.2	2.683	2	5	
7	4	8	6.2	1.643	7	3	
8	0	3	1.4	1.517	1	3	
9	7	15	10.2	3.421	10	6.5	

**Figure 6:**
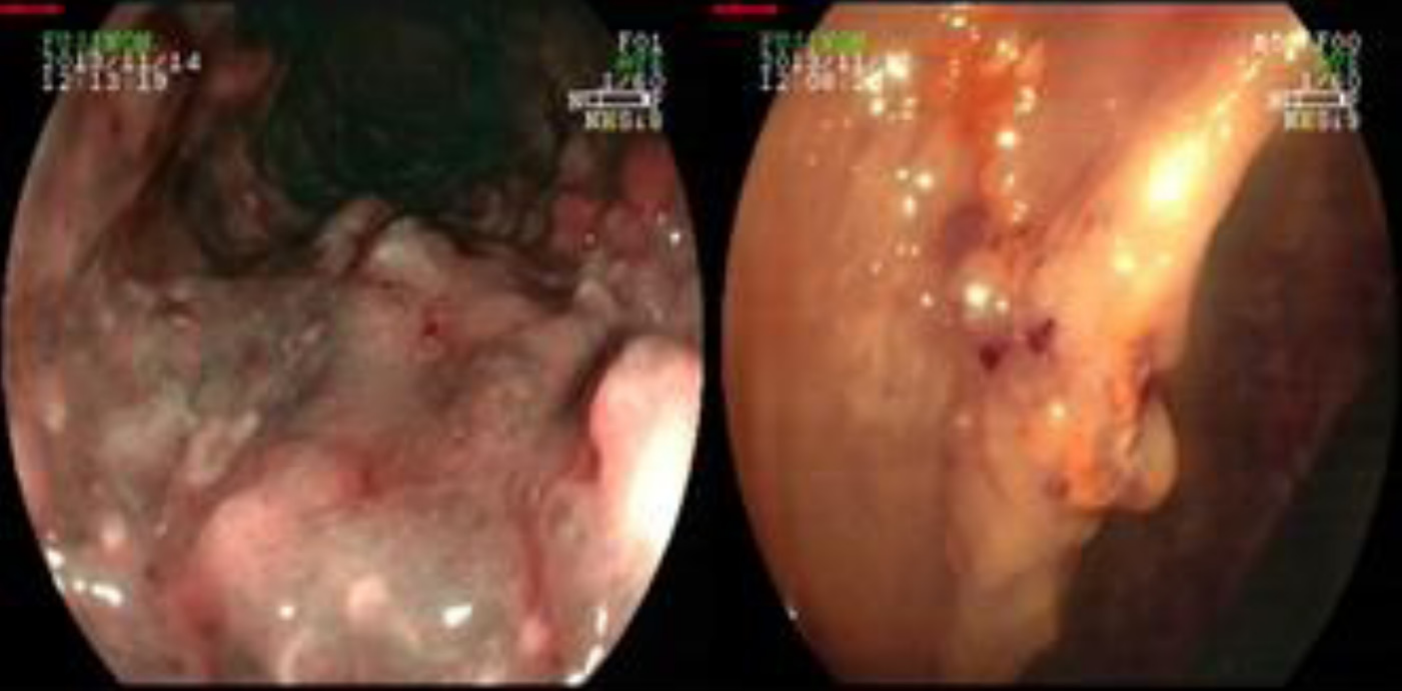
Dysplasia-associated lesion or mass-like lesions as depicted with the eighth flexible spectral imaging color enhancement (FICE) channel (left) and the ninth FICE channel (right).

In addition to polypoid lesions, target biopsy revealed 3 dysplasia-associated lesion or mass (DALM)-like lesions. The round pit pattern dominated in the surface assessment. In another lesion, polypoid lesions were raised from the irregular mucosa on the pili in the transverse colon (Figure 6). The third lesion was hardly distinguished by standard imaging, but was visible with FICE. This lesion was slightly raised from the irregular mucosa in a larger area. No dysplasia was observed in the biopsies of the 3 lesions.

### FICE images in mild to moderate colitis

**Figure 7:**
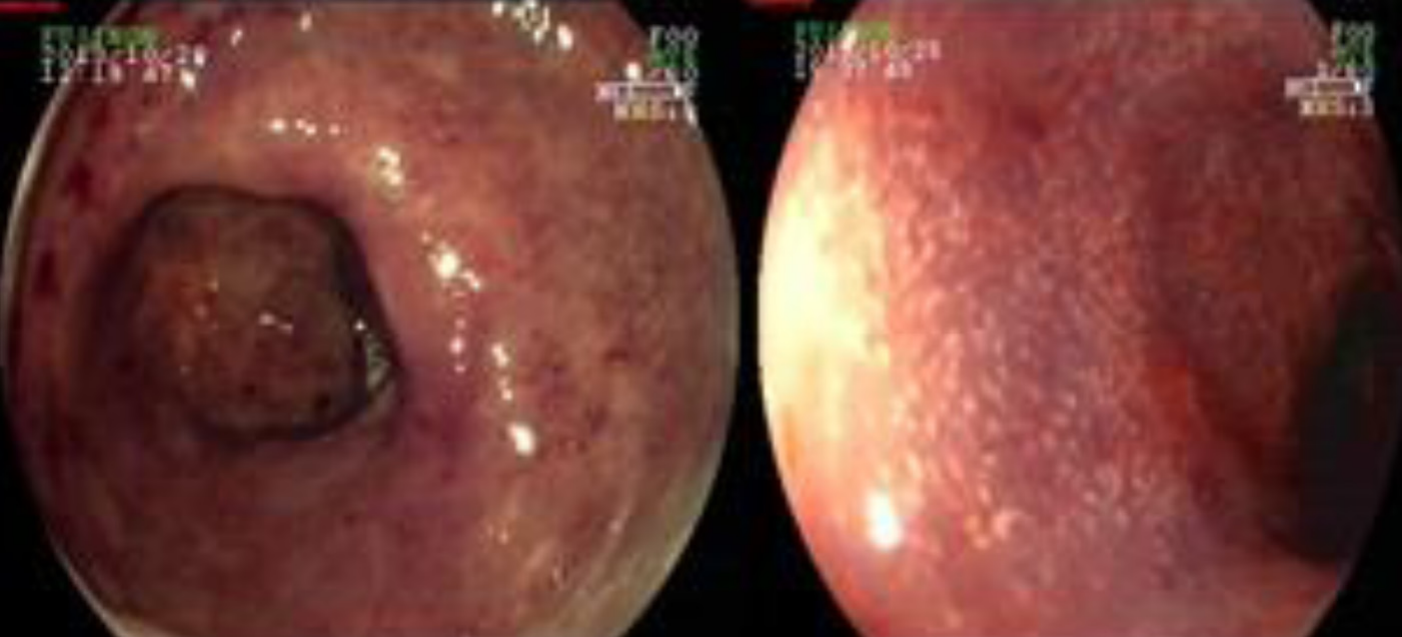
Active colitis as depicted with the third flexible spectral imaging color enhancement (FICE) channel (left) and the ninth FICE channel (right).

The segments endoscopically observed using standard imaging as hyperemic, edematous, and superficially ulcerated were also imaged with FICE, and biopsies were obtained. Clinical and laboratory findings associated with active colitis were detected endoscopically in 7 of 18 patients in remission. Fourteen segments (7 in the rectum, 4 in the sigmoid, 2 in the transverse colon, and 1 in the descending colon) of active colitis were evaluated using FICE. The biopsies showed inflammatory cells, crypt hyperplasia, and crypt distortion. The presence and limits of inflammation were clearly observed using FICE in the areas where the capillary network was erased. Crypt hyperplasia and crypt hypertrophy were detected in the mucosa biopsy, in which white dotted crypt structures predominated (Figure 7).

The scores of 50 images of five patients with active inflamed colon mucosa significantly differed across 10 FICE channels (*P* < 0.001). The median values were 9 (IQR, 8.5), 9 (IQR, 6), and 9 (IQR, 7.5) in the second, third, and ninth channel, respectively. There was no significant difference among the three channels (*P* > 0.05) (Table 6).

**Table 6:** Rating scores awarded to images obtained with different flexible spectral imaging color enhancement (FICE) channels for colitis mucosa

Channels	Minimum	Maximum	Mean	Standard deviation	Median	Interquartile range	*P*
0	0	2	0.8	1.095	0	2	<0.001
1	0	11	4.2	4.087	3	6	
2	0	18	10.4	4.93	9	8.5	
3	5	13	8.6	3.209	9	6	
4	5	1	0.2	0.447	0	0.5	
5	0	6	2.2	2.49	2	4.5	
6	0	4	2	1.581	2	3	
7	0	6	2.4	2.302	2	4	
8	0	7	2	2.915	1	4.5	
9	4	15	9.2	4.147	9	7.5	

In conclusion, the scores of 150 images from 15 different areas significantly differed across 10 channels of FICE (*P* < 0.001). The channels that best visualized the mucosal vascular structure were the second and ninth channel. The scores of these two channels did not significantly differ (Table 6).

### Comparison of FICE images and standard images

The second and ninth channel, which were the best FICE channels, were compared with standard images (Table 8). FICE images obtained higher scores than standard images, but the difference was not significant. There was no difference in different image types either.

**Table 8:** The comparison of second and ninth flexible spectral imaging color enhancement (FICE) channels with standard images

	Channel	Mean	Standard deviation	Median	Interquartile range	*P*
Normal (n = 35)	FICE2	1.17	0.822	1.00	1	0.059
	FICE9	0.89	0.796	1.00	1	
Active (n = 35)	FICE2	1.31	0.932	1.00	1	0.710
	FICE9	1.37	0.770	1.00	1	
Polyp (n = 35)	FICE2	1.06	0.998	1.00	0	0.294
	FICE9	0.91	0.818	1.00	1	
Total (n = 105)	FICE2	1.18	0.918	1.00	1	0.158
	FICE9	1.06	0.818	1.00	1	

## DISCUSSION

In this study, FICE was not significantly better than the standard procedure at dysplasia screening, but it was effective at detecting diminutive polyps and at evaluating surface patterns without using magnification. FICE might contribute to the assessment of inflammation severity in patients with UC in clinical remission. To our knowledge, this is the first study investigating the contribution of FICE to CRC screening in UC.

Previous studies have evaluated early colorectal neoplastic lesions and colon polyps using FICE. Liu et al (12) compared standard colonoscopy with CE and all FICE channels with magnification. The lesion recognition rates were 91.1% with standard colonoscopy, 100% with indigocarmine CE, and 99.1% with FICE. Moreover, FICE was more successful in detecting polyps smaller than 5 mm (16-18).

FICE, especially with HRME, was found to have high sensitivity and specificity in neoplastic differentiation of colon polyps according to surface patterns (18). A prospective study by Pohl et al (18) showed that FICE better visualized the pit pattern and vascular structures than standard colonoscopy. The sensitivities of standard colonoscopy, KE, and FICE techniques with low magnification were 76.4%, 91%, and 89.9%, with specificities of 65.6%, 62.7%, and 73.8%, respectively. The sensitivity (84.3%, 95.5%, and 96.6%, respectively) and specificity (64%, 73.8%, and 80.3%, respectively) increased when high magnification was used (13,18). Our study neither compared FICE with another endoscopic technique nor used magnification.

The surface patterns of colon polyps can be examined without the use of magnification. A study assessing 151 colorectal polyps compared the fourth FICE channel, CE, and standard endoscopy without the use of magnification (19). The sensitivity of FICE in distinguishing neoplastic from nonneoplastic lesions was 89.4%, with a specificity of 89.2%. The sensitivity of CE was 96.8%, and the specificity was 89.2% (19). The study concluded that FICE was more practical than CE and that it was superior to standard endoscopy in distinguishing malignant lesions without magnification. In this study, neoplastic and nonneoplastic colon polyps could also be differentiated despite the small number of patients just by evaluating the surface patterns with FICE without magnification.

FICE was reported to have a high detection rate of especially diminutive polyps. Togashi et al evaluated 107 polyps smaller than 5 mm (89 neoplastic and 27 nonneoplastic) using standard colonoscopy, FICE (third channel), and indigocarmine CE (17). The detection rate of standard endoscopy was 75%, with a sensitivity of 71% and a specificity of 81%. Interestingly, the detection rates of FICE and CE were 87% and 96%, with sensitivities of 93% and 90% and specificities of 70% and 74%, respectively (17). Other studies have also shown that FICE increased the detection rates of polyps smaller than 5 mm compared with standard colonoscopy (44.4% vs 21.5%, *P* = 0.0006) (16). In our study, three of the lesions detected as dysplasia were diminutive polyps. The surface pattern of only one lesion could not be assessed. However, the other two lesions could be clearly detected by FICE compared with standard endoscopy. Thus, FICE might be useful in the detection of flat lesions and diminutive polyps in screening colonoscopies for cancer in patients with UC.

FICE and NBI are similar techniques, both being highly effective at detailed depiction of mucosal surface and at evaluating vascular structures because of the combined magnified and high-resolution colonoscopy. NBI clearly distinguished mucosal vascular pattern (MVP) in determining inflammation severity in UC (20). Two MVP subtypes were identified: “similar to honeycomb” and “irregular” (20). In active UC, intramucosal capillary network disappeared, and round-crypt or villous mucosal structures were prominent. Crypt atrophy or distortion was histopathologically detected in biopsies taken from the villous mucosal structure. The authors concluded that NBI with magnification can be used for *in vivo* evaluation of mucosal inflammation in inactive UC (20). Another study used NBI to image 157 colorectal segments. Sixty segments had normal MVP and in 97 had irregular MVP. Of the 97 irregular segments, MVP was clearly distinguished in 44 segments. The group with no MVP compared with the irregular MVP group significantly more frequently had acute inflammatory infiltration and goblet cell depletion (21). Although some classifications defined vascular pattern in colorectal lesions and Barrett esophagus using FICE, no study or classification has assessed the use of FICE in UC. It is difficult to clearly distinguish vascular patterns using FICE without magnification. Thinner and smaller vascular structures are better identified by NBI than by FICE. In our study, when normal colon mucosa identified by standard endoscopy was imaged with FICE, capillary network was viewed as light brown and submucosal vessels were viewed as dark brown, and the surface structure and capillary network could be clearly distinguished. When hyperemic, edematous, granular mucosa identified by standard endoscopy was imaged with FICE, intramucosal capillary network completely disappeared, while white dotted crypt structures were prominent. FICE imaging is particularly effective at determining the limits of inflammation and at distinguishing ulcerated structures. Acute inflammation findings, crypt abscess, crypt hyperplasia, and crypt distortion were detected in the biopsies taken from these areas. Our study did not find a vascular pattern because colitis mucosa was imaged without magnification.

A study has demonstrated that the fourth, seventh, and tenth channel best diagnosed stomach and duodenum mucosa (22). Some studies evaluated colorectal lesions with the third, fourth, fifth, sixth, seventh, eighth, and ninth FICE channel. Two endoscopists found staining CE and all FICE channels to better assess mucosal and vascular pattern than white light endoscopy (WLE) in 560 colon segments of 10 patients. The second and fourth FICE channels best visualized the vascular pattern, while the fourth channel best assessed mucosal and vascular pattern together (23). Teixeira et al also found the fourth channel to best depict the capillary network when distinguishing neoplastic and nonneoplastic lesions (24). We found that the second, sixth, and ninth channel best depicted the vascular structure of the normal colon mucosa. The third, seventh, and ninth, channels best visualized polyps, and the second, third, and ninth channel best visualized colitis mucosa. When we compared the best three channels among themselves, the ninth was significantly better in visualizing polyp images than the third channel.

The best method for dysplasia screening in UC has so far been staining CE. Studies could not demonstrate the superiority of NBI, one of the virtual CE techniques, to staining CE. In our study, the second and ninth FICE channel were superior to both other channels and standard WLE imaging, but no significant difference was observed. FICE imaging and Kudo classification were in agreement with histopathological findings.

Despite the limitation of a small number of patients, the current study can be used as a guide for FICE channel selection in studies on UC. FICE was not superior to standard imaging in dysplasia screening in UC. However, further studies comparing FICE with other techniques are necessary. FICE was observed to be effective in the detection of diminutive polyps and evaluation of surface pattern without using magnification. Studies using FICE should be planned in order to determine the presence and severity of inflammation in UC patients in clinical remission. New colonoscopic techniques integrated with artificial intelligence are expected to improve the daily colonoscopy practice in the future.

**Table 7:** Rating scores awarded to images obtained with different flexible spectral imaging color enhancement (FICE) channels for all assessed conditions

Channels	Minimum	Maximum	Mean	Standard deviation	Median	Interquartile range	*P*
0	0	9	2.13	3.021	2	3	<0.001
1	0	11	4.07	3.283	3	2	
2	0	18	9.07	5.338	9	9	
3	0	13	4.8	3.764	5	5	
4	0	5	0.8	1.474	0	1	
5	0	6	1.2	1.699	0	2	
6	0	14	5.47	4.658	4	9	
7	0	8	3.13	2.825	2	5	
8	0	7	1.13	1.959	0	2	
9	0	15	10.2	3.299	10	5	
